# 2-Phenyl­imidazolium hemi(benzene-1,3-dicarboxyl­ate) monohydrate

**DOI:** 10.1107/S160053681102681X

**Published:** 2011-07-16

**Authors:** Wen-Yu Zhang, Zhi-Hua Zhu, Jian-Shi Du

**Affiliations:** a China–Japan Union Hospital, Jilin University, Changchun 130033, People’s Republic of China

## Abstract

The asymmetric unit of the title compound, C_9_H_9_N_2_^+^·0.5C_8_H_4_O_4_^−^·H_2_O, contains one 2-phenyl­imidazolium cation, half a benzene-1,3-dicarboxyl­ate anion and one water mol­ecule. In the crystal, components are connected by N—H⋯O and O—H⋯O hydrogen-bonding inter­actions into a three-dimensional network.

## Related literature

For related 2-phenyl­imidazolium structures, see: Xia *et al.* (2009 ▶); Zhang *et al.* (2007 ▶). 
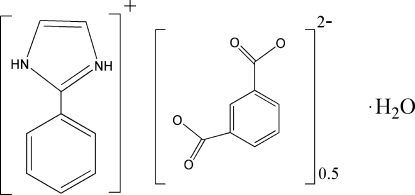


## Experimental

### 

#### Crystal data

C_9_H_9_N_2_^+^·0.5C_8_H_4_O_4_^−^·H_2_O
*M* *_r_* = 245.25
Monoclinic, 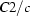

*a* = 17.092 (5) Å
*b* = 7.152 (4) Å
*c* = 20.322 (5) Å
β = 108.449 (3)°
*V* = 2356.5 (16) Å^3^
*Z* = 8
Mo *K*α radiation
μ = 0.10 mm^−1^
*T* = 293 K
0.23 × 0.19 × 0.18 mm

#### Data collection

Oxford Diffraction Gemini R Ultra diffractometer
Absorption correction: multi-scan (*CrysAlis RED*; Oxford Diffraction, 2006 ▶) *T*_min_ = 0.57, *T*_max_ = 0.74
4898 measured reflections
2400 independent reflections
1762 reflections with *I* > 2σ(*I*)
*R*_int_ = 0.052

#### Refinement

*R*[*F* ^2^ > 2σ(*F* ^2^)] = 0.067
*wR*(*F* ^2^) = 0.194
*S* = 1.10
2400 reflections
172 parameters
3 restraints
H atoms treated by a mixture of independent and constrained refinement
Δρ_max_ = 0.42 e Å^−3^
Δρ_min_ = −0.38 e Å^−3^

### 

Data collection: *CrysAlis CCD* (Oxford Diffraction, 2006 ▶); cell refinement: *CrysAlis CCD*; data reduction: *CrysAlis RED* (Oxford Diffraction, 2006 ▶); program(s) used to solve structure: *SHELXS97* (Sheldrick, 2008 ▶); program(s) used to refine structure: *SHELXL97* (Sheldrick, 2008 ▶); molecular graphics: *SHELXTL* (Sheldrick, 2008 ▶); software used to prepare material for publication: *SHELXTL*.

## Supplementary Material

Crystal structure: contains datablock(s) global, I. DOI: 10.1107/S160053681102681X/bt5571sup1.cif

Structure factors: contains datablock(s) I. DOI: 10.1107/S160053681102681X/bt5571Isup2.hkl

Additional supplementary materials:  crystallographic information; 3D view; checkCIF report

## Figures and Tables

**Table 1:** Hydrogen-bond geometry (Å, °)

*D*—H⋯*A*	*D*—H	H⋯*A*	*D*⋯*A*	*D*—H⋯*A*
N1—H1⋯O1*W*	0.86	1.88	2.725 (3)	169
N2—H2⋯O2^i^	0.86	1.89	2.731 (3)	167
O1*W*—H*W*11⋯O1	0.85 (1)	1.92 (1)	2.718 (3)	156 (3)
O1*W*—H*W*12⋯O2^ii^	0.85 (1)	2.01 (1)	2.858 (2)	176 (3)

Symmetry codes: (i) 
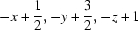
; (ii) 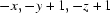.

**Fig. 1.**
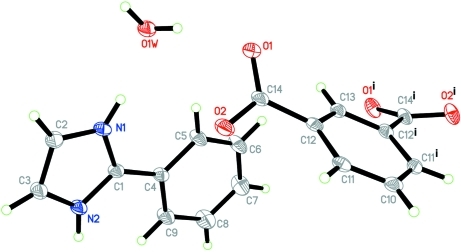
The structure of (I), showing the atomic numbering scheme. Displacement ellipsoids are drawn at the 30% probability level. Symmetry code: (i) -x, y, 0.5 - z.

## supplementary crystallographic information

### Comment

The 2-phenylimidazole easily forms supramolecular structures with anions. The 2-phenylimidazolium nitrate structure has been reported as a hydrate (Xia *et al.*, 2009) and a hemihydrate (Zhang *et al.*, 2007). In this work, we report the synthesis and structure of the hemi-benzene-1,3-dicarboxylate hydrate, namely C_9_H_9_N_2_^+.^0.5(C_8_O_4_H_4_)^-.^H_2_O.

The asymmetric unit of the title compound consists of one 2-phenylimidazolium cation and half a benzene-1,3-dicarboxylate, and one water molecule (Fig. 1). In the structure, there exist N—H···O and O—H···O hydrogen-bonding interactions (Table I).

### Experimental

A mixture of 2-phenylimidazole (0.5 mmol), benzene-1,3-dicarboxylic acid (0.5 mmol) and H_2_O (5 ml) was mixed. After seven days, colorless crystals were obtained at room temperature.

### Refinement

All H atoms on C and N atoms were positioned geometrically (N—H = 0.86 Å and C—H = 0.93 Å) and refined as riding, with *U*_iso_(H)=1.2*U*_eq_(carrier). H atoms of the water molecules were located in a difference Fourier map and refined as riding with the O—H and H···H distance restraints of 0.85±0.01 and 1.35±0.01 Å, respectively.

### Crystal data

**Table:**

C_9_H_9_N_2_^+^·0.5C_8_H_4_O_4_^−^·H_2_O	*F*(000) = 1032
*M**_r_* = 245.25	*D*_x_ = 1.383 Mg m^−^^3^
Monoclinic, *C*2/*c*	Mo *K*α radiation, λ = 0.71073 Å
Hall symbol: -C 2yc	Cell parameters from 2400 reflections
*a* = 17.092 (5) Å	θ = 2.1–26.4°
*b* = 7.152 (4) Å	µ = 0.10 mm^−^^1^
*c* = 20.322 (5) Å	*T* = 293 K
β = 108.449 (3)°	Block, colorless
*V* = 2356.5 (16) Å^3^	0.23 × 0.19 × 0.18 mm
*Z* = 8	

### Data collection

**Table:**

Oxford Diffraction Gemini R Ultra diffractometer	2400 independent reflections
Radiation source: fine-focus sealed tube	1762 reflections with *I* > 2σ(*I*)
graphite	*R*_int_ = 0.052
Detector resolution: 10.0 pixels mm^-1^	θ_max_ = 26.4°, θ_min_ = 2.1°
ω scans	*h* = −20→21
Absorption correction: multi-scan (*CrysAlis RED*; Oxford Diffraction, 2006)	*k* = −7→8
*T*_min_ = 0.57, *T*_max_ = 0.74	*l* = −25→18
4898 measured reflections	

### Refinement

**Table:**

Refinement on *F*^2^	Primary atom site location: structure-invariant direct methods
Least-squares matrix: full	Secondary atom site location: difference Fourier map
*R*[*F*^2^ > 2σ(*F*^2^)] = 0.067	Hydrogen site location: inferred from neighbouring sites
*wR*(*F*^2^) = 0.194	H atoms treated by a mixture of independent and constrained refinement
*S* = 1.10	*w* = 1/[σ^2^(*F*_o_^2^) + (0.0998*P*)^2^ + 0.3011*P*] where *P* = (*F*_o_^2^ + 2*F*_c_^2^)/3
2400 reflections	(Δ/σ)_max_ < 0.001
172 parameters	Δρ_max_ = 0.42 e Å^−^^3^
3 restraints	Δρ_min_ = −0.38 e Å^−^^3^

### Special details

**Table:**

Geometry. All e.s.d.'s (except the e.s.d. in the dihedral angle between two l.s. planes) are estimated using the full covariance matrix. The cell e.s.d.'s are taken into account individually in the estimation of e.s.d.'s in distances, angles and torsion angles; correlations between e.s.d.'s in cell parameters are only used when they are defined by crystal symmetry. An approximate (isotropic) treatment of cell e.s.d.'s is used for estimating e.s.d.'s involving l.s. planes.
Refinement. Refinement of *F*^2^ against ALL reflections. The weighted *R*-factor *wR* and goodness of fit *S* are based on *F*^2^, conventional *R*-factors *R* are based on *F*, with *F* set to zero for negative *F*^2^. The threshold expression of *F*^2^ > σ(*F*^2^) is used only for calculating *R*-factors(gt) *etc*. and is not relevant to the choice of reflections for refinement. *R*-factors based on *F*^2^ are statistically about twice as large as those based on *F*, and *R*- factors based on ALL data will be even larger.

### Fractional atomic coordinates and isotropic or equivalent isotropic displacement parameters (Å^2^)

**Table:**

	*x*	*y*	*z*	*U*_iso_*/*U*_eq_	
C1	0.24852 (14)	0.4967 (3)	0.50929 (13)	0.0382 (5)	
C2	0.20650 (15)	0.4816 (4)	0.60149 (13)	0.0467 (6)	
H2A	0.1734	0.4629	0.6294	0.056*	
C3	0.28571 (15)	0.5424 (4)	0.62248 (13)	0.0486 (7)	
H3	0.3173	0.5726	0.6676	0.058*	
C4	0.24990 (14)	0.4906 (3)	0.43856 (12)	0.0382 (6)	
C5	0.17948 (15)	0.4488 (4)	0.38343 (13)	0.0492 (7)	
H5	0.1305	0.4193	0.3919	0.059*	
C6	0.18210 (17)	0.4509 (4)	0.31638 (14)	0.0604 (8)	
H6	0.1348	0.4238	0.2797	0.072*	
C7	0.2544 (2)	0.4929 (5)	0.30332 (16)	0.0674 (9)	
H7	0.2561	0.4949	0.2580	0.081*	
C8	0.32373 (19)	0.5315 (5)	0.35745 (16)	0.0677 (9)	
H8	0.3726	0.5589	0.3485	0.081*	
C9	0.32284 (16)	0.5307 (4)	0.42428 (14)	0.0538 (7)	
H9	0.3708	0.5569	0.4604	0.065*	
C10	0.0000	1.1129 (5)	0.2500	0.0448 (8)	
H10	0.0000	1.2429	0.2500	0.054*	
C11	0.00630 (13)	1.0179 (3)	0.31049 (12)	0.0408 (6)	
H11	0.0122	1.0838	0.3512	0.049*	
C12	0.00388 (12)	0.8237 (3)	0.31058 (11)	0.0363 (5)	
C13	0.0000	0.7293 (4)	0.2500	0.0368 (7)	
H13	0.0000	0.5993	0.2500	0.044*	
C14	0.00529 (13)	0.7151 (4)	0.37391 (12)	0.0421 (6)	
N1	0.18476 (11)	0.4532 (3)	0.53205 (10)	0.0417 (5)	
H1	0.1374	0.4135	0.5064	0.050*	
N2	0.31015 (12)	0.5509 (3)	0.56483 (10)	0.0427 (5)	
H2	0.3581	0.5859	0.5643	0.051*	
O1	−0.03238 (11)	0.5623 (2)	0.36545 (9)	0.0539 (5)	
O2	0.04493 (10)	0.7828 (3)	0.43224 (8)	0.0541 (5)	
O1W	0.03320 (10)	0.3051 (3)	0.46633 (9)	0.0501 (5)	
HW11	0.0013 (19)	0.383 (4)	0.4395 (15)	0.114 (15)*	
HW12	0.0109 (18)	0.273 (4)	0.4965 (12)	0.079 (10)*	

### Atomic displacement parameters (Å^2^)

**Table:**

	*U*^11^	*U*^22^	*U*^33^	*U*^12^	*U*^13^	*U*^23^
C1	0.0242 (11)	0.0425 (12)	0.0480 (13)	−0.0003 (9)	0.0114 (10)	0.0017 (10)
C2	0.0323 (13)	0.0656 (16)	0.0466 (14)	−0.0023 (11)	0.0187 (11)	0.0042 (12)
C3	0.0337 (13)	0.0716 (18)	0.0409 (13)	−0.0074 (11)	0.0125 (11)	−0.0009 (12)
C4	0.0233 (11)	0.0438 (12)	0.0472 (13)	0.0031 (9)	0.0109 (10)	0.0003 (10)
C5	0.0250 (12)	0.0703 (17)	0.0496 (14)	0.0002 (11)	0.0082 (10)	−0.0061 (12)
C6	0.0316 (14)	0.093 (2)	0.0512 (15)	0.0030 (13)	0.0050 (12)	−0.0105 (14)
C7	0.0512 (18)	0.104 (2)	0.0505 (16)	0.0047 (16)	0.0209 (14)	−0.0061 (15)
C8	0.0388 (15)	0.111 (2)	0.0629 (18)	−0.0018 (15)	0.0302 (15)	−0.0041 (16)
C9	0.0282 (13)	0.0802 (19)	0.0537 (15)	−0.0025 (12)	0.0140 (12)	−0.0036 (13)
C10	0.0234 (15)	0.0411 (17)	0.066 (2)	0.000	0.0092 (15)	0.000
C11	0.0221 (11)	0.0498 (14)	0.0488 (14)	−0.0019 (9)	0.0086 (10)	−0.0085 (10)
C12	0.0191 (10)	0.0475 (13)	0.0419 (13)	−0.0031 (9)	0.0090 (9)	−0.0037 (10)
C13	0.0241 (15)	0.0393 (17)	0.0448 (17)	0.000	0.0076 (13)	0.000
C14	0.0221 (10)	0.0626 (15)	0.0414 (13)	−0.0059 (10)	0.0097 (9)	−0.0002 (11)
N1	0.0225 (10)	0.0525 (12)	0.0501 (12)	−0.0045 (8)	0.0117 (9)	0.0002 (9)
N2	0.0270 (10)	0.0589 (13)	0.0417 (11)	−0.0072 (9)	0.0101 (9)	−0.0014 (9)
O1	0.0406 (10)	0.0668 (12)	0.0494 (10)	−0.0176 (9)	0.0074 (8)	0.0070 (8)
O2	0.0379 (10)	0.0851 (13)	0.0381 (10)	−0.0188 (9)	0.0105 (8)	−0.0028 (8)
O1W	0.0341 (9)	0.0674 (13)	0.0502 (11)	−0.0059 (9)	0.0152 (9)	0.0019 (9)

### Geometric parameters (Å, °)

**Table:**

C1—N2	1.335 (3)	C8—H8	0.9300
C1—N1	1.348 (3)	C9—H9	0.9300
C1—C4	1.446 (3)	C10—C11^i^	1.378 (3)
C2—N1	1.356 (3)	C10—C11	1.378 (3)
C2—C3	1.356 (3)	C10—H10	0.9300
C2—H2A	0.9300	C11—C12	1.389 (3)
C3—N2	1.364 (3)	C11—H11	0.9300
C3—H3	0.9300	C12—C13	1.387 (3)
C4—C5	1.393 (3)	C12—C14	1.497 (3)
C4—C9	1.396 (3)	C13—C12^i^	1.387 (3)
C5—C6	1.378 (4)	C13—H13	0.9300
C5—H5	0.9300	C14—O1	1.252 (3)
C6—C7	1.377 (4)	C14—O2	1.261 (3)
C6—H6	0.9300	N1—H1	0.8600
C7—C8	1.365 (4)	N2—H2	0.8600
C7—H7	0.9300	O1W—HW11	0.848 (10)
C8—C9	1.363 (4)	O1W—HW12	0.848 (10)
			
N2—C1—N1	106.5 (2)	C8—C9—H9	120.0
N2—C1—C4	126.35 (19)	C4—C9—H9	120.0
N1—C1—C4	127.1 (2)	C11^i^—C10—C11	121.0 (3)
N1—C2—C3	107.0 (2)	C11^i^—C10—H10	119.5
N1—C2—H2A	126.5	C11—C10—H10	119.5
C3—C2—H2A	126.5	C10—C11—C12	120.0 (2)
C2—C3—N2	106.9 (2)	C10—C11—H11	120.0
C2—C3—H3	126.5	C12—C11—H11	120.0
N2—C3—H3	126.5	C13—C12—C11	118.6 (2)
C5—C4—C9	118.6 (2)	C13—C12—C14	119.6 (2)
C5—C4—C1	121.6 (2)	C11—C12—C14	121.9 (2)
C9—C4—C1	119.8 (2)	C12—C13—C12^i^	121.7 (3)
C6—C5—C4	120.2 (2)	C12—C13—H13	119.1
C6—C5—H5	119.9	C12^i^—C13—H13	119.1
C4—C5—H5	119.9	O1—C14—O2	124.4 (2)
C7—C6—C5	120.3 (3)	O1—C14—C12	117.9 (2)
C7—C6—H6	119.8	O2—C14—C12	117.8 (2)
C5—C6—H6	119.8	C1—N1—C2	109.7 (2)
C8—C7—C6	119.4 (3)	C1—N1—H1	125.1
C8—C7—H7	120.3	C2—N1—H1	125.1
C6—C7—H7	120.3	C1—N2—C3	109.82 (19)
C9—C8—C7	121.4 (3)	C1—N2—H2	125.1
C9—C8—H8	119.3	C3—N2—H2	125.1
C7—C8—H8	119.3	HW11—O1W—HW12	107.3 (16)
C8—C9—C4	120.0 (3)		

Symmetry codes: (i) −*x*, *y*, −*z*+1/2.

### Hydrogen-bond geometry (Å, °)

**Table:**

*D*—H···*A*	*D*—H	H···*A*	*D*···*A*	*D*—H···*A*
N1—H1···O1W	0.86	1.88	2.725 (3)	169
N2—H2···O2^ii^	0.86	1.89	2.731 (3)	167
O1W—HW11···O1	0.85 (1)	1.92 (1)	2.718 (3)	156 (3)
O1W—HW12···O2^iii^	0.85 (1)	2.01 (1)	2.858 (2)	176 (3)

Symmetry codes: (ii) −*x*+1/2, −*y*+3/2, −*z*+1; (iii) −*x*, −*y*+1, −*z*+1.
